# Screening for Retinopathy of Prematurity Through Utilization a Pediatric Retinal Camera at Jim Pattison Children’s Hospital: A Vision for Improved Care

**DOI:** 10.1177/2333794X211039642

**Published:** 2021-09-30

**Authors:** Malshi Karunatilake, Sibasis Daspal, Veronica Mugarab Samedi, Shehla Rubab

**Affiliations:** 1 University of Saskatchewan, Saskatoon, SK, Canada; 2 Royal University Hospital Children’s Services, Saskatoon, SK, Canada

**Keywords:** Retinopathy of prematuirty, digital retinal imaging, screening

## Abstract

Retinopathy of Prematurity (ROP) is a vascular proliferative disorder of preterm infants, with increased disease severity and incidence occurring with lower gestational age and birth weight. An alternate approach to ROP screening with wide-field digital retinal imaging helps with the early detection of ROP, especially during the pandemic.

## Background

Retinopathy of Prematurity (ROP) is a vascular proliferative disorder of preterm infants, with increased disease severity and incidence occurring with lower gestational age and birth weight.^4^ With the higher survival rates of premature infants due to advancing perinatal care, incidence of ROP will continue to rise.^17^ ROP can lead to serious adverse outcomes such as retinal detachment, poor visual acuity, and blindness.^4^ Therefore, early detection is key to management of ROP which may include prompt laser photocoagulation, as early as within 72 hours.^10^

It is recommended that all infants with a birth weight of ≤1500 g or gestational age ≤30 weeks as well as selected infants with a birth weight of 1500 to 2000 g or gestational ages >30 weeks who are deemed to be at high risk of ROP as per a neonatologist or pediatrician. These screening recommendations are supported by institutions such as the Canadian Pediatric Society,^10^ American Academy of Ophthalmology (AAO),^6^ and American Academy of Pediatrics (AAP).^6^

ROP screening involves a dilated eye examination by an experienced ophthalmologist with a binocular indirect ophthalmoscope (BIO).^6^ Depending on the severity of ROP and gestational age, infants may require multiple follow up examinations. Despite the increasing need for ROP screening, the number of ophthalmologists available at bed side can vary due to issues such as time constraints.^17^

Although important, this examination comes at a cost—discomfort to the infant along with various negative physiological effects. The International Evidence-Based Group for Neonatal Pain has listed ROP examination as one of the diagnostic painful procedures performed in the NICU.^2^ Reported effects include tachycardia, bradycardia, increase in blood pressure, apnea, and desaturation episodes.^11,16^ The effects could be secondary to the oculocardiac reflex^20^ as well as the mydriatic eye drops.^16^

Studies have shown that supportive interventions such as anesthetic eye drops,^12^ swaddling, and oral sucrose^7^ can decrease neonatal stress during BIO examination, though the strength of the effect varied.^14,18^

## Ethical Approval and Informed Consent

Ethical Approval was not applicable, because this manuscript is review article and does not contain any data with human or animal subjects.

## BIO Versus WFDRI

An alternate approach to ROP screening involves the use of wide-field digital retinal imaging (WFDRI). This mode of non-contact imaging can be performed by trained personnel other than an ophthalmologist,^3^ which would drastically contribute to efficiency of image capture and increase the volume of screened infants at a time. A prospective cohort study by Prakalpakorn et al^15^ showed that non-contact cameras are well tolerated and less stressful to the infant. Moral-Pumarega et al^13^ found less pain with WFDRI at 30 seconds after the examination.

**Figure 1.**
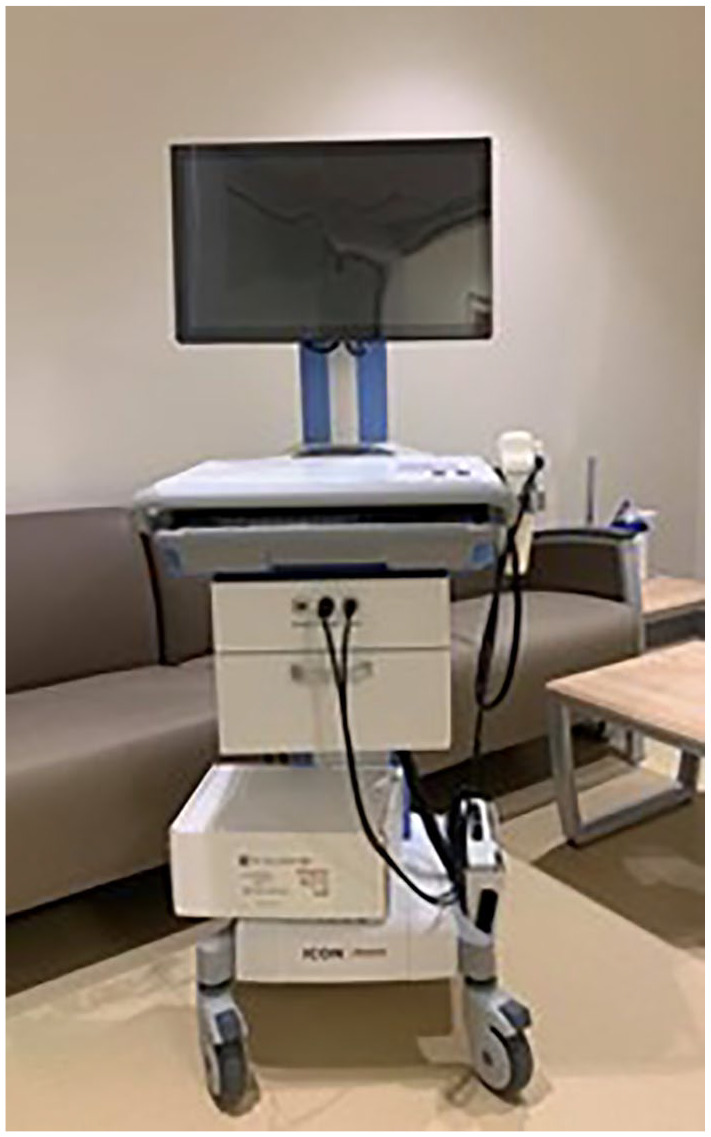
Phoenix ICON at JPCH.

One such retinal camera is the Phoenix ICON Paediatric Retinal Camera, a recent addition to the NICU at JPCH (Figure 1).

## Brief Overview of Phoenix ICON Paediatric Retinal Camera

This lightweight hand-held camera allows for a wide field-of-view of the retina. It also enables white light and fluorescein angiography with reduced injected light levels which facilitates patient comfort. The ensemble also includes a lightweight LED light, touchscreen display, large work surface, full-size keyboard, trackball as well as a motorized vertical height adjustment. Its software which meets healthcare security requirements, makes it effortless to capture, review, and report the images.^8^

## Current Utilization at JPCH

Since the arrival of ICON at Jim Pattison Children’s Hospital (JPCH) in June 2020, 37 infants have been screened for ROP using the Phoenix ICON.

The images are being captured by trained healthcare personnel who are already a part of the infant’s care team; this has decreased unnecessary patient contact that could occur with repeated exams by multiple ophthalmologists, thereby reducing risk of infection and patient discomfort.

Further, ophthalmologists’ availability does not always coincide with an optimal time for an eye exam since the infant may be distressed, feeding, or receiving treatment. By utilizing Phoenix ICON, it has been possible capture retinal images when the infant is already soothed and comfortable, along with increased efficiency of the image capture process.

Phoenix ICON also allows comparison of images, thus, allowing easy monitoring of ROP progression. Unlike in BIO, it enables revision of images by multiple ophthalmologists without needing repeated eye examinations.

## Phoenix ICON and the COVID Pandemic

ICON has been particularly helpful during the COVID pandemic given that it is a non-contact mode of imaging.

The Phoenix ICON ensemble also has a hand piece holster with a built-in soaking cup and soak timer which facilitates disinfection and helps maintain a disinfection audit log.^8^

Since it has been used by healthcare personnel that are already involved in the infant’s care, it minimizes the infant’s exposure to novel contacts.

## What’s Next?

The transfer of at-risk infants from level III NICUs to remote ones are often delayed for their eye exam to occur—this has been associated to increased health care costs, visual morbidity, and inconvenience for families.^1^

Therefore, the use of easy-to-use, non-invasive, digital retinal imaging that could be implemented in rural sites begs the question for its role for remote ROP screening. In fact, teleophthalmology programs that utilize digital retinal cameras have been explored around the world, including Canada.^19^ Ells et al^5^ conducted a pilot longitudinal cohort study based in Alberta that utilized digital retinal photography with remote image reading and identified its potential role for using telemedicine in ROP screening. The Ontario Telemedicine for Retinopathy of Prematurity (ONTROP) has developed a program that has paired digital retinal imaging with a 2-way audio-video connection for ROP screening.^1^ A cost analysis of ONTROP by Isaac et al^9^ further supports this initiative given the lower average total cost per eye exam of infants in the telemedicine group compared to control. This calls for continuing exploration of wide-field retinal imaging as a part of teleophthalmology initiatives around the country in order to provide safe, efficient ROP screening that is reasonably accessible to infants in remote communities.

## Final Thoughts

The diagnostic value of a wide-field digital retinal imaging system along with its role in efficiency, safety, and patient comfort has made it a valuable asset to the NICU at JPCH. The Phoenix ICON has been particularly ideal during the pandemic since it requires minimal contact with the patient. Its role in rural ROP screening should be further explored when developing a teleophthalmology program that strive for improved level of patient care.
