# Corrigendum to Screening for Retinopathy of Prematurity Through Utilization a Pediatric Retinal Camera at Jim Pattison Children’s Hospital: A Vision for Improved Care

**DOI:** 10.1177/2333794X221079522

**Published:** 2022-02-10

Karunatilake M, Daspal S, Samedi VM, Rubab S. Screening for Retinopathy of Prematurity Through Utilization a Pediatric Retinal Camera at Jim Pattison Children’s Hospital: A Vision for Improved Care. *Global Pediatric Health*; 2021, Vol. 8: 1-4. 10.1177/2333794X211039642

The authors missed to mention Assistant Professor Vasudha Erraguntla in the authorship whereas her contribution satisfies the requirements of authorship.

The revised order of the authors and Author Contribution statement are as follows:

1. Malshi Karunatilake, 2. Sibasis Daspal, 3. Veronica Mugarab Samedi, 4. Shehla Rubab, 5. Vasudha Erraguntla

**Author Contributions**

MK: Contributed to conception and design; Contributed to analysis; Drafted the manuscript; revised the manuscript. SD:Contributed to conception and design; Contributed to acquisition and interpretation; critically revised the manuscript; gave final approval; Agrees to be accountable for all aspects of work ensuring integrity and accuracy. VMS: Contributed to conception and design; critically revised the manuscript; gave final approval; Agrees to be accountable for all aspects of work ensuring integrity and accuracy. SR: Contributed to acquisition and interpretation, gave final approval. VE: Contributed to acquisition and interpretation of data; critically revised the manuscript; gave final approval; Agrees to be accountable for all aspects of work ensuring integrity and accuracy.

